# Association between BDNF levels and suicidal behaviour: a systematic review and meta-analysis

**DOI:** 10.1186/s13643-015-0179-z

**Published:** 2015-12-30

**Authors:** Rebecca B. Eisen, Stefan Perera, Laura Banfield, Rebecca Anglin, Luciano Minuzzi, Zainab Samaan

**Affiliations:** MiNDS Neuroscience Graduate Program, McMaster University, 1280 Main Street West, Hamilton, ON L8S 4L8 Canada; Health Research Methodology Graduate Program, McMaster University, 1280 Main Street West, Hamilton, ON L8S 4L8 Canada; Department of Clinical Epidemiology and Biostatistics, McMaster University, 1280 Main Street West, Hamilton, ON L8S 4L8 Canada; Health Sciences Library, McMaster University, 1280 Main Street West, Hamilton, ON L8S 4L8 Canada; Department of Psychiatry and Behavioural Neurosciences, McMaster University, 1280 Main St. West, Hamilton, ON L8S 4L8 Canada; Department of Medicine, McMaster University, 1280 Main Street West, Hamilton, ON L8S 4L8 Canada; Women’s Health Concerns Clinic, St. Joseph’s Healthcare Hamilton, 50 Charlton Avenue East, Hamilton, ON L8N 4A6 Canada; Population Genomics Program, Chanchlani Research Centre, McMaster University, 1280 Main Street West, Hamilton, ON L8S 4L8 Canada; Peter Boris Centre for Addiction Research, St. Joseph’s Healthcare Hamilton, 100 West 5th Street, Hamilton, ON L8P 3R2 Canada

**Keywords:** Suicide, Attempted suicide, Suicidal ideation, Brain-derived neurotrophic factor, Systematic review, Meta-analysis

## Abstract

**Background:**

Suicidal behaviour is a complex phenomenon with a multitude of risk factors. Brain-derived neurotrophic factor (BDNF), a protein crucial to nervous system function, may be involved in suicide risk. The objective of this systematic review is to evaluate and summarize the literature examining the relationship between BDNF levels and suicidal behaviour.

**Methods:**

A predefined search strategy was used to search MEDLINE, EMBASE, PsychINFO, and CINAHL from inception to December 2015. Studies were included if they investigated the association between BDNF levels and suicidal behaviours (including completed suicide, attempted suicide, or suicidal ideation) by comparing BDNF levels in groups with and without suicidal behaviour. Only the following observational studies were included: case-control and cohort studies. Both clinical- and community-based samples were included. Screening, data extraction, and risk of bias assessment were conducted in duplicate.

**Results:**

Six-hundred thirty-one articles were screened, and 14 were included in the review. Three studies that assessed serum BDNF levels in individuals with suicide attempts and controls were combined in a meta-analysis that showed no significant association between serum BDNF and suicide attempts. The remaining 11 studies were not eligible for the meta-analysis and provided inconsistent findings regarding associations between BDNF and suicidal behaviour.

**Conclusions:**

The findings of the meta-analysis indicate that there is no significant association between serum BDNF and attempted suicide. The qualitative review of the literature did not provide consistent support for an association between BDNF levels and suicidal behaviour. The evidence has significant methodological limitations.

**Systematic review registration:**

PROSPERO CRD42015015871

**Electronic supplementary material:**

The online version of this article (doi:10.1186/s13643-015-0179-z) contains supplementary material, which is available to authorized users.

## Background

Suicide is a growing public health concern. Worldwide, over 800,000 people die by suicide every year, and the numbers are increasing [1]. Suicide affects not only the individual but the family, community, and society in which it occurs. Non-fatal suicidal behaviours, which refer to a complex set of thoughts, plans, and acts intended to end one’s life, are significant risk factors for completed suicide and occur 10–20 times more often than completed suicide [1, 2].

A multitude of factors are thought to contribute to the risk of suicidal behaviour, including internal (biological and psychological) and external (social and environmental) factors. Examples of internal risk factors include psychiatric disorders, substance-use disorders, chronic illness, and demographic variables (such as older age and female sex) [3]. External risk factors can include unmarried status, unemployment, and a lack of social support [2, 3]. Most suicidal behaviour occurs in the context of a psychiatric disorder (90 % of attempted or completed suicides), but most individuals with psychiatric disorders never attempt suicide [3, 4]. In addition, many cases of suicide cannot be explained by the conventional risk factors that have been proposed by research and clinical observations. Consequently, there is a need to identify predictors of suicidal behaviour beyond the known risk factors [2].

Recent research has focused on biological markers of suicide risk, such as genetic variants and circulating proteins [5]. One such protein is brain-derived neurotrophic factor (BDNF), a member of the neurotrophin family of proteins. BDNF is found in the brain and throughout the body in the bloodstream [6]. It is crucial to a number of neural processes, such as neurogenesis, neuroplasticity, and neurotransmission [6, 7].

Altered BDNF levels may play a role in the pathogenesis of suicidal behaviour by resulting in long-term changes in the brain that can lead to neuropsychological deficits. A number of studies have shown that changes in brain structure and function may be associated with depression, stress, and suicidal behaviour. These changes include reductions in neuron cell number, density, and size, as well as decreased cortical thickness and changes in synaptic circuitry [8–11]. Other studies demonstrate cognitive deficits in stress and depression [12]. This evidence supports a new hypothesis that links the pathogenesis of suicidal behaviour and depression to altered neural plasticity, which impairs the brain’s ability to respond appropriately to environmental stimuli [13, 14]. It is hypothesized that pathological changes in BDNF levels are distally responsible for these neuropsychological deficits associated with depression, stress, and suicide [6].

It is also possible that short-term changes in BDNF levels may be involved in suicidal behaviour pathogenesis. There is evidence that BDNF levels can undergo short-term variations in response to external stimuli. Serum BDNF levels have been shown to increase following a 3-month reduced-calorie diet [15] and endurance training [16]. Antidepressant treatment in depressed individuals normalizes low levels of BDNF [17]. Alcohol and tobacco use have also been linked to altered levels of BDNF; excessive drinkers tend to have lower serum BDNF levels, and current smokers tend to have higher serum BDNF levels [18]. These and other variables may explain variations in BDNF level between and within individuals over time. These factors may also be related to risk of suicidal behaviour and could explain how BDNF might be related to suicidal behaviour in a proximal manner. However, there is no conclusive evidence linking short-term changes in BDNF to suicidal behaviour.

While BDNF is primarily produced in the central nervous system, it also expressed in peripheral tissue in smooth muscle cells, endothelial cells, endocrine cells, and immune cells [18]. It has been shown to cross the blood-brain barrier, and blood levels of BDNF are reflective of brain levels [19]. Brain levels of BDNF can be measured in postmortem brain tissue, and circulating levels can be measured in the blood (serum or plasma) and cerebrospinal fluid (CSF) of living individuals.

Altered central and peripheral BDNF levels have been implicated in both depression [20–22] and stress [23–26], both of which are risk factors for suicidal behaviour [6]. Furthermore, altered BDNF levels have been linked to suicidal behaviour in postmortem brain studies [27, 28]. Clinical studies have shown reduced peripheral BDNF levels in both the serum and plasma of suicidal individuals [29–31]. While this is a growing area of research, the relationship between BDNF levels and suicidal behaviour remains unclear, as relatively few studies have explored this relationship. In addition, since some of these studies have examined recent suicidal behaviour while others examined lifetime suicidal behaviour, it is uncertain whether BDNF is related to suicide in a distal or proximal manner. To date, there has not been a systematic review undertaken to summarize the literature.

This paper aims to systematically evaluate and summarize the existing literature relating BDNF levels (including central and peripheral levels) to suicidal behaviour (including completed suicide, attempted suicide, and suicidal ideation) in adult populations. Based on our current understanding of BDNF and its role in brain structure and function, it is expected that low BDNF levels will be associated with suicidal behaviour in studies of both central and peripheral BDNF levels.

## Methods

### Search strategy

The protocol for this systematic review was published previously [32]. This systematic review follows the Preferred Reporting Items for Systematic Reviews and Meta-Analyses (PRISMA) guidelines as well as the Meta-analysis of Observational Studies in Epidemiology (MOOSE) guidelines (see Additional file 1 for PRISMA checklist). An a priori-defined search strategy was developed with the help of an experienced health sciences librarian (LB) and was used to search the following databases from inception until December 2015: PubMed/MEDLINE, PsychINFO, EMBASE, and CINAHL. The search strategy can be found in the published protocol. One amendment was made to the original search strategy. Because many cases of suicidal behaviour involve overdose of substances, two search terms (“self poison” and “overdose”) were added to capture studies of those behaviours. An example of the search strategy for MEDLINE is presented in Table 1. The grey literature was searched for previously published theses using the ProQuest Dissertations and Theses A&I database. The reference lists of included articles were scanned manually.

**Table 1:** Medline Search Strategy

Database	Search Strategy
MEDLINE (n=124)	1. exp Suicide/ 2. suicid*.mp. 3. exp Self-Injurious Behavior/ 4. (self harm* or self inflict* or self injur* or self wound* or self mutilat* or self poison* or overdose).mp. 5. automutilat*.mp 6. 1 or 2 or 3 or 4 or 5 7. brain derived neurotrophic factor.mp. or Brain-Derived Neurotrophic Factor/ 8. bdnf.mp 9. 7 or 8 10. 6 and 9

### Inclusion and exclusion criteria

This review included observational studies that investigated associations between levels of BDNF (central or peripheral, including postmortem brain tissue, cerebral spinal fluid, and blood) and suicidal behaviours (including completed suicide, attempted suicide, and suicidal ideation) in adult populations (aged 18 and older). Studies of both clinical and community-based samples were included. No demographic limitations were applied apart from age.

### Screening and data extraction

**Fig. 1:**
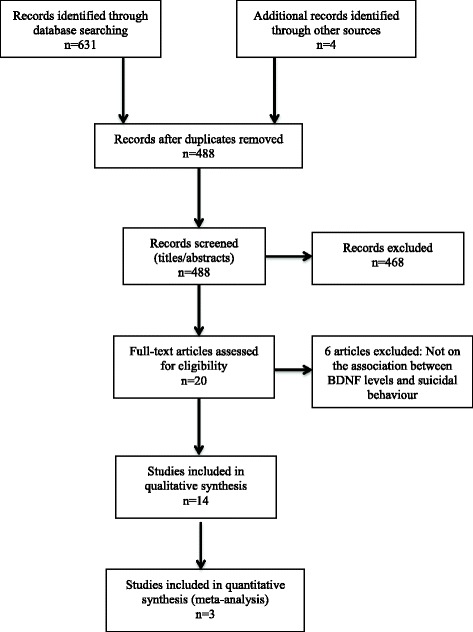
PRISMA flow diagram. Studies selected for inclusion with number of studies included after each stage of the screening process

Two raters (RE and SP) screened the articles identified by the literature search. Articles were screened first by title, then by abstract for full-text review. Studies that met the inclusion criteria upon full-text review were identified for data extraction. For articles that were excluded, reasons for exclusion were documented (see Fig. 1). Discrepancies at any point in the screening process were resolved by discussion, and in cases of disagreement, a third rater (ZS) was consulted. The Kappa statistic was used to calculate inter-rater agreement [33].

**Fig. 2:**
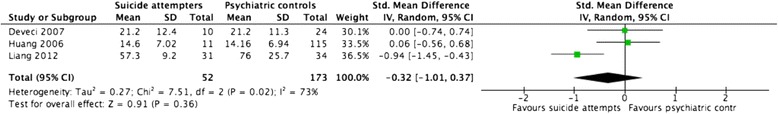
Serum BDNF in attempted suicide. Legend: Forest plot representing the differences in serum BDNF level between individuals with suicide attempts and psychiatric controls

Two independent raters (RE and SP) performed the data extraction in duplicate. A pre-established, pilot-tested data extraction form was used. The following information was obtained from each study: study information (title, author, publication year, journal name, location of study), study setting and design, description of sample and comparison groups, sample size, definition and measurement of suicidal behaviour, type of BDNF sample, statistical methods, mean BDNF levels and standard deviations, and limitations. Four authors were contacted for further information, and one responded with numerical data not presented in the article. One article was published in Mandarin [34], so a fluent Mandarin speaker assisted with the determination of its eligibility and data extraction. Risk of bias in included studies was assessed in duplicate using an adapted version of the Newcastle-Ottawa Scale, which assesses for risk of selection bias, performance bias, detection bias, and information bias [35].

## Results

### Search results

The database search retrieved 631 records. After the removal of duplicates, 488 titles were screened, and 438 records were excluded. An additional 30 were excluded upon the review of the abstracts. Twenty full-text articles were assessed for eligibility. Of these, 14 were included in this review (see Fig. 1 for PRISMA flow diagram). Inter-rater agreement for the title, abstract, and full-text screening was 0.59, 0.71, and 0.91, respectively, corresponding to fair, good, and excellent agreement [36].

### Study characteristics

The characteristics of the included studies are summarized in Table 2. Twelve of the included articles were case-control studies, and two were cross-sectional studies. Four of the studies were postmortem studies that measured BDNF in brain tissue samples from individuals who had died by suicide. The remaining nine studies were clinical studies, eight of which investigated blood levels of BDNF (serum or plasma) in participants who attempted suicide, and one of which investigated cerebrospinal fluid (CSF) levels of BDNF in individuals who experienced suicidal ideation.

**Table 2:** Study Characteristics

Author (year)	Study design and sample groups	Participants (*n*)	Definition of suicidal behaviour	Type of BDNF measurement (units)	Mean (SD) BDNF levels	p-value (difference between groups)	Variables adjusted for
Postmortem Brain Studies							
Banerjee et al. (2013)	Case–control Cases of suicide vs. non-psychiatric healthy controls	Suicide: 21 Control: 19	Completed suicide	Brain – hippocampus (pg/ml)	Suicide: 19.5^a^ Control: 44^a^	<0.001	None
Dwivedi et al. (2003)	Case–control Cases of suicide vs. non-psychiatric controls	Suicide: 27 Control: 21	Completed suicide	Brain – PFC, hippocampus (optical density)	PFC Suicide + MDD: 0.94 (0.22) Suicide + other psychiatric disorder: 0.88 (0.28) Control: 1.61 (0.39) Hippocampus Suicide + MDD: 1.04 (0.20) Suicide + other psychiatric disorder: 1.03 (0.22) Control: 1.71 (0.44)	<0.001 <0.001	None
Karege et al. (2005)	Case–control Cases of suicide vs. non-psychiatric controls	Suicide: 30 Control: 24	Completed suicide	Brain – PFC, hippocampus, entorhinal cortex (ng/g)	Hippocampus Drug-free MDD: 17.7 (2.9) Drug-free others: 16.8 (3.1) Drug-treated MDD: 23.3 (2.2) Drug-free controls: 24.5 (3.6) PFC Drug-free MDD: 13.8 (2.6) Drug-free others: 12.7 (2.6) Drug-treated MDD: 17.9 (2.9) Drug-free controls: 17.5 (3.0) Entorhinal cortex Drug-free MDD: 14.1 (2.1) Drug-free others: 13.4 (2.4) Drug-treated MDD: 12.9 (2.3) Drug-free controls: 13.4 (2.5)	<0.001 <0.002 Not significant	PMI, age
Maheu et al. (2013)	Case–control Depressed individuals (divided into suicide victims vs. non-suicide deaths) vs. non-psychiatric controls	Depressed suicide (12) Depressed non-suicide (10) Control (14)	Completed suicide	Brain – BLA, CeA (optical density)	BLA Depressed suicide: 1.19 (0.80) Depressed non-suicide: 1.67 (1.42) Control: 1.08 (0.34) CeA Depressed suicide: 0.95 (0.20) Depressed non-suicide: 1.87 (1.30) Control: 0.97 (0.16)	Not significant Not significant	PMI, brain pH, age
CSF Studies							
Martinez et al. (2012)	Case–control Depressed patients vs. non-depressed patients	Depressed (18) Non-depressed (25)	Suicidal ideation	CSF (pg/ml)	NA	Correlation between BDNF concentration and SSI score: r = 0.62, P = 0.033, n = 12	None
Serum BDNF Studies							
Deveci et al. (2007)	Case–control Suicide attempters vs. non-suicidal MDD patients vs. healthy controls	Suicide (10) Non-suicidal MDD (24) Healthy controls (26)	Suicide attempt	Serum (ng/ml)	Suicide: 21.2 (12.24) Non-suicidal MDD: 21.2 (11.3) Control: 31.4 (8.8)	Suicide vs. healthy controls: 0.004 Suicide vs. MDD: not significant	None
Grah et al. (2014)	Case–control MDD patients vs. PD patients vs. AD patients vs. healthy controls	Suicide (96) Control (106) ^b^	Suicide attempt	Serum (ng/ml)	RDD ^c^ Suicide: 11.8 (8.88–14.73) Control: 12.8 (10.83–14.78) PD Suicide: 10.7 (7.04–14.26) Control: 15.7 (10.29–21.02) AD Suicide: 12.6 (9.63–15.58) Control: 15.4 (12.04–18.77)	0.07 0.003 0.009	Age, sex, therapy
Huang & Lee (2006)	Case–control Schizophrenic patients vs. healthy controls	Suicidal schizophrenic (11) Non-suicidal schizophrenic (115) Healthy control (96)	Suicide attempt	Serum (ng/ml)	Suicidal schizophrenic: 14.60 (7.02) Non-suicidal schizophrenic: 14.16 (6.94)	0.841	None
Liang et al. (2012)	Case–control Depressed patients (with or without suicide attempt) vs. healthy controls	Suicidal depressed (31) Non-suicidal depressed (34) Healthy control (30)	Suicide attempt, suicidal ideation	Serum (ng/ml)	Suicidal depressed: 57.3 (9.2) Non-suicidal depressed: 76.0 (25.7) Healthy control: 113.8 (44.4)	Suicide attempt: <0.01 Suicidal ideation: <0.01	None
Park et al. (2014)	Cross-sectional MDD patients	Suicidal MDD (18) Non-suicidal MDD (33)	Suicide attempt	Serum (ng/ml)	Suicidal MDD: 21.93 (24.71) Non-suicidal MDD: 24.71 (7.7)	0.3	None
Pinheiro et al. (2014)	Cross-sectional Postpartum women	History of suicide attempts (12) No suicide history (178)	Suicide attempt, suicidal ideation	Serum (ng/ml)	Suicide: 2.11 (1.42) Control: 2.37 (1.26)	0.6 Linear regression of PPAD and suicide risk: −0.912 (−1.73– -0.09) p = 0.029	Previous psychiatric treatment, stressful life events during pregnancy
Plasma BDNF Studies							
Kim et al. (2007)	Case–control Suicidal depressed patients vs. non-suicidal depressed patients vs. healthy controls	Suicidal depressed (32) Non-suicidal depressed (32) Control (30)	Suicide attempt	Plasma (pg/ml)	Suicidal depressed: 430.5 (397.0) Non-suicidal depressed: 875.80 (663.02) Control: 889.4 (611.3)	0.002	None
Lee et al. (2007)	Case–control Depressed patients vs. healthy controls	Suicidal depressed (28) Non-suicidal depressed (49) Control (95)	Suicide attempt	Plasma (pg/ml)	Suicidal depressed: 386.61 (362.39) Non-suicidal depressed: 689.66 (404.65) Control: 819.20 (347.05)	<0.001	None
Lee & Kim (2009)	Case–control Suicidal depressed patients vs. non-suicidal depressed patients vs. healthy controls	Suicidal depressed (20) Non-suicidal depressed (20) Control (20)	Suicide attempt	Plasma ^d^ (pg/ml)	Suicidal depressed: 713.04 (236.56) Non-suicidal depressed: 693.98 (347.84) Control: 709.05 (172.12)	0.971	None

^a^Mean BDNF values estimated from inspection of graph

^b^3 diagnosis groups: Recurrent depressive disorder, personality disorder, adjustment disorder

^c^Medians and interquartile ranges presented instead of means and SD

^d^BDNF was measured in 3 types of sample: platelet-rich plasma, platelet-poor plasma, and platelets. Results are presented here for only the platelet-poor plasma measurement

*Abbreviations*: *BDNF* brain-derived neurotrophic factor, *SD* standard deviation, *MDD* major depressive disorder, *PFC* prefrontal cortex, *PMI* postmortem interval, *BLA* basolateral amygdala, *CeA* central nucleus of the amygdala, *RDD* recurrent depressive disorder, *PD* personality disorder, *AD* adjustment disorder, *PPAD* postpartum affective disorder, *CSF* cerebrospinal fluid

### Risk of bias assessment

The modified version of the Newcastle-Ottawa Scale contains seven questions that fall under four domains: methods for selecting study participants (selection bias), methods to control for confounding (performance bias), statistical methods (detection bias), and methods of exposure and outcome assessment (information bias). Assessing the included studies using the modified Newcastle-Ottawa Scale revealed a number of common sources of risk of bias. Nearly all of the studies (12/14) had samples sizes that were small, likely resulting in insufficient power to detect meaningful differences in mean BDNF level between groups. Most of the studies (10/14) compared groups of between 20 and 30 participants, though some groups were as small as 10 participants. Another significant source of high risk of bias was the lack of adjustment for confounding variables. While some studies matched participants on age and/or sex in an attempt to reduce confounding, only four of the studies adjusted for any variables in their analyses. Finally, twelve of the fourteen studies used statistical methods that are inappropriate for observational studies. Univariate analyses were used to compare mean BDNF levels between groups. Only two studies [37, 38] performed a regression analysis to investigate the relationship between BDNF levels and suicidal behaviour (see Additional file 2 for a table displaying the scores from the risk of bias assessment).

### Postmortem brain studies of completed suicide

Four of the included studies examined protein levels of BDNF in the brains of postmortem subjects [27, 28, 39, 40] (see Table 2). These studies employed a case-control design to compare protein levels in individuals who died by suicide with levels in non-suicide deaths. The studies have a combined total of 90 cases and 88 controls. Dwivedi et al. (2003) [27] used the Western blot technique to determine the protein levels of BDNF in prefrontal cortex (PFC) and hippocampal samples from 27 individuals who died by suicide and 21 non-psychiatric control subjects. They found significant differences in BDNF expression between the groups in both brain regions, with lower levels in the individuals who died by suicide. The authors assert that these differences were unrelated to psychiatric diagnosis or other measured variables (postmortem interval, brain pH, age, and sex). In a similar study, Karege et al. (2005) [28] compared BDNF levels in the ventral prefrontal cortex, hippocampus, and entorhinal cortex between 30 individuals who died by suicide and 24 non-psychiatric controls. The group of individuals who died by suicide was subdivided into three groups by diagnosis and toxicology: untreated depressed, untreated other psychiatric disorder, and drug-treated depressed. The enzyme-linked immunosorbent assay (ELISA) technique was used to quantify BDNF in the tissue samples. Significantly reduced BDNF levels were found in both of the non-treated suicide groups compared to the non-suicide group, in the PFC (*p* < 0.002) and hippocampus (*p* < 0.001), but not in the drug-treated suicide group. No significant differences were found in the entorhinal cortex of any group. The third study to look at postmortem hippocampal levels of BDNF was conducted by Banerjee et al. (2013) [39]. They also employed the ELISA method to compare BDNF levels between 21 individuals who died by suicide and 19 non-psychiatric controls. A significant difference was found between the groups, with reduced BDNF levels in individuals who died by suicide (*p* < 0.001). The final study to examine brain levels of BDNF focused exclusively on the amygdala. Maheu et al. (2013) [40] measured BDNF levels in the basolateral amygdala (BLA) and central amygdala (CeA) of depressed individuals (22 and 25, respectively), and 14 healthy controls. Eleven of the depressed subjects from which the BLA was sampled died of suicide, and twelve of the depressed subjects from which the CeA was sampled died of suicide. No significant differences were found between mean BDNF levels in individuals who died by suicide compared to controls.

### Cerebrospinal fluid BDNF levels and attempted suicide

Only one study examined the association between BDNF in the CSF and suicidal behaviour. Martinez et al. [41] compared levels of pro-inflammatory and “resiliency” proteins (among them BDNF) between 18 depressed individuals and 25 healthy controls. While the mean BDNF levels were not presented or compared between suicidal and non-suicidal groups, the correlation between BDNF concentration and score on the Scale for Suicidal Ideation (SSI) was calculated for 12 participants. A significant positive correlation was found between BDNF concentration and SSI score (*r* = 0.62, *p* = 0.033).

### Serum BDNF levels and attempted suicide

Two of the included studies were cross-sectional studies that investigated serum levels of BDNF in clinical sample populations. These studies collectively assessed 241 individuals. Park et al. (2014) [42] conducted a pilot study relating serum BDNF levels to illness severity, suicide attempts, and central serotonin activity in depressed patients. The patients were stratified into subgroups based on their history of suicide attempts; 18 had a history of suicide attempts and 33 did not. Mean BDNF levels did not differ significantly between the two groups (*p* = 0.3). The other cross-sectional study was conducted by Pinheiro et al. (2012) [38] in postpartum women. Of the 190 women included, 12 had a history of suicide attempts. No significant difference was found between mean BDNF levels in this group compared to the women with no history of suicide attempt (*p* = 0.6). However, in women with postpartum affective disorder (*n* = 29), suicide risk, as measured with the suicidality section of the Mini International Neuropsychiatric Interview (MINI), was significantly associated with lower BDNF levels (*p* = 0.02).

The remaining four studies of serum BDNF and suicidal behaviour were case-control in design. The studies include a combined total of 148 cases and 335 controls. Two studies [29, 34] compared individuals with suicide attempts to both psychiatric and healthy controls. Deveci et al. (2007) [29] recruited 10 individuals with suicide attempts, 24 non-suicidal depressed individuals, and 26 healthy controls. Serum BDNF levels were found to be significantly lower in both the suicide group and the depressed group compared to the healthy control group (*p* = 0.004). However, there was no significant difference between BDNF levels in the suicide and depressed groups. Liang et al. (2012) [34] conducted a study comparing BDNF levels in depressed patients, with and without a history of suicide attempts, and healthy controls. The sample consisted of 31 depressed individuals with suicide attempts, 34 depressed individuals without suicide attempts, and 30 healthy controls. Serum BDNF levels were significantly different among the three groups, with the lowest levels in the suicide group (*p* < 0.01). Among the 65 depressed individuals, BDNF levels were negatively correlated with scores on the Self-rating Idea of Suicide Scale (SIOSS) (*p* < 0.01) [34].

The final two studies of serum BDNF levels focused on specific psychiatric disorders. Huang and Lee (2006) [43] measured BDNF levels in a group of 126 patients with schizophrenia, 11 of which had a history of suicide attempts. No significant difference in mean BDNF level was found (*p* = 0.841). In a study by Grah et al. (2014) [37], associations between BDNF levels and suicidal behaviour were explored in patients suffering from depression, personality disorders, and adjustment disorders. The study included 51 patients with recurrent depressive disorder, 26 of which were suicidal; 59 patients with personality disorders, 33 of which were suicidal, 62 patients with adjustment disorders, 37 of which were suicidal; and 60 healthy controls. Significantly lower BDNF levels were found in those with suicide attempts in the personality disorder and adjustment disorder groups (*p* = 0.003, *p* = 0.009, respectively), but not in the depressed group.

A meta-analysis was performed using the results of three case-control studies that compared serum BDNF levels between suicide attempters and psychiatric controls [29, 34, 43] (Figure 2). These studies were selected for inclusion in the meta-analysis based on their similar study designs (all case-control studies), definitions of suicidal behaviour (attempted suicide), and comparison groups (psychiatric controls). A random-effects model was used. The pooled estimate revealed a standardized mean difference (SMD) of −0.32 (95 % CI −1.01 to 0.37), which corresponds to a small effect size according to Cohen’s criteria [44]. However, this estimate was not significant (*p* = 0.36) and was associated with substantial heterogeneity (*I*^2^ = 73 %, *p* = 0.02).

### Plasma BDNF levels and attempted suicide

Three case-control studies measured plasma levels of BDNF in depressed individuals with and without a history of suicidal behaviour [30, 31, 45]. These studies collectively assessed 80 cases and 246 controls. Kim et al. (2007) [30] compared 32 depressed patients hospitalized for recent suicide attempts to 32 hospitalized non-suicidal depressed patients and 30 healthy controls. They found significantly reduced plasma BDNF levels in suicide attempters compared to both control groups (*p* = 0.009, *p* = 0.008, respectively). Lee et al. (2007) [31] measured plasma BDNF levels in 77 hospitalized depressed patients (subdivided into 28 with a suicide attempt and 49 without a suicide attempt) and 95 healthy controls. This study also found a significant difference between BDNF levels in suicidal vs. non-suicidal individuals, with lower levels in the suicidal depressed group compared to the non-suicidal depressed group. Lee and Kim (2009) [45] conducted a study similar to Kim et al.’s [30] in which 20 hospitalized depressed individuals with recent suicide attempts were compared to 20 hospitalized non-suicidal depressed patients and 20 healthy controls. BDNF was measured in platelet-rich plasma, platelet-poor plasma, and platelets. In all three types of sample, BDNF levels were significantly lower in depressed patients (suicidal and non-suicidal) compared to healthy controls, but no significant differences were found between suicidal and non-suicidal groups.

## Discussion

This systematic review aimed to evaluate and summarize the existing literature on associations between BDNF levels and suicidal behaviour. The 14 studies included in this review describe comparisons of mean BDNF levels between groups of individuals with and without suicidal behaviour (see Table 2). The definitions of suicidal behaviour, the methods of measuring BDNF level, and the sample populations, vary widely. The studies differ in their findings and methodological quality, producing an unclear picture of the relationship between BDNF levels and suicidal behaviour.

### Postmortem brain studies of completed suicide

The postmortem studies of BDNF levels and completed suicide have examined multiple brain regions, including the hippocampus, prefrontal cortex, entorhinal cortex, and amygdala. Three studies [27, 28, 39] measured BDNF protein levels in the hippocampus and all found significant associations with completed suicide, suggesting that individuals who die by suicide have lower levels of BDNF. Two of those studies [27, 28] also measured BDNF levels in the PFC and found significant inverse associations with completed suicide. In the other brain regions studied, the entorhinal cortex [28] and the amygdala [40], no significant differences were found.

Of the four studies of brain BDNF levels in people who died by suicide, only one, Maheu et al. [40], included both psychiatric and non-psychiatric controls. The other three studies compared individuals who died by suicide to non-psychiatric controls. BDNF levels are altered in depression and other psychiatric disorders. In addition, most suicides occur in the context of a psychiatric disorder, suggesting that individuals with a psychiatric illness are a particularly vulnerable population for suicidal behaviour. In order to determine the association between BDNF and suicidal behaviour, a comparison group should be derived from a psychiatric population, in addition to healthy controls. Maheu et al.’s study was the only postmortem study that did not find a significant association between BDNF and suicide. The differences found in the other three studies could have resulted from altered BDNF levels associated with psychiatric disorders rather than suicidal behaviour. Therefore, one should be cautious when interpreting the results of the other studies, as their significant findings may not represent an association between BDNF and suicide.

Another important factor to consider is the effect of psychotropic medications on BDNF levels. Only one study, Karege et al. [28], explored this variable. They separated the group of people who died by suicide by toxicology by determining the presence of therapeutic drugs in the body. They found differences in BDNF levels among the groups. They found a significant association between BDNF level and suicidal behaviour when comparing drug-free suicide completers to controls, but not when comparing drug-treated suicide completers to controls. Future studies should investigate and control for the effects of antidepressants and other medications on BDNF levels in postmortem suicide deaths.

Postmortem studies are subject to a number of limitations, making it difficult to draw sound conclusions from them. Factors prior to death can affect the integrity of the brain’s morphology and biochemical content [46]. Depending on the cause and manner of death, changes in blood oxygenation, brain perfusion, and acid-base balance can have varying effects on the brain and on the variables of interest in postmortem studies. Different methods of suicide can produce different effects on the brains. Postmortem interval (PMI), the time between death and freezing or fixing of the brain tissue, also influences the quality of the tissue. PMI can have complex and unknown effects on the outcome measure being studied [46]. Only two of the postmortem studies of BDNF and suicide adjusted for confounding variables in their analyses [28, 40]. Both Maheu et al. and Karege et al. adjusted for PMI and age, and Maheu et al. also adjusted for brain pH. Future postmortem studies should assess and control for factors that influence the integrity of the brain tissue samples.

Bearing in mind these limitations, one can cautiously conclude from the existing evidence that an association may exist between brain levels of BDNF (particularly in the hippocampus and prefrontal cortex) and completed suicide. However, additional studies with larger samples and psychiatric comparators are needed to confirm this association.

### Cerebrospinal fluid BDNF levels and attempted suicide

The one study of CSF levels of BDNF and suicidal behaviour, by Martinez et al. [41], found that increased levels of BDNF were significantly associated with higher levels of suicidal ideation. This finding is contradictory to the hypothesis that lower levels of BDNF are associated with suicidal behaviour. However, the sample size for the analysis was very small (12 participants), and the analysis did not adjust for confounding factors. Additional well-powered studies are necessary to explore this association. At this point, no conclusions can be drawn regarding the association of CSF levels of BDNF and suicidal behaviour.

### Serum BDNF levels and attempted suicide

The six studies of serum BDNF levels and suicidal behaviour vary widely in their findings. Of the studies that looked at attempted suicide, three found significant associations and three did not. Two of the studies also investigated suicidal ideation and found a significant relationship with BDNF levels.

The limitations of the studies’ methodologies could have resulted in biased estimates and inconsistent findings. The sample sizes were generally modest, with case groups ranging from 10 to 31 participants. None of the studies adjusted for confounding variables in their analyses, even though observational studies are inherently prone to influences by many confounding variables. Of the six studies of serum BDNF and suicidal behaviour, only two performed adjusted analyses. Grah et al. [37] adjusted for age, sex, and therapy, while Pinheiro et al. [38] adjusted for previous psychiatric treatment and stressful life events during pregnancy.

Another factor that could account for the inconsistent findings among studies is the variation in time periods between suicide attempts and BDNF measurement. While Deveci et al.’s study included individuals who were hospitalized for a recent suicide attempt, other studies included individuals with a lifetime history of suicide attempts. In studies including participants with a lifetime history of suicide attempts, the BDNF measurement could have occurred within weeks, months, or years of the suicide attempt, and the precise time interval is neither known nor accounted for in the analysis. Because BDNF levels vary over time in response to a number of external factors, the BDNF measurements in these studies may not represent the levels at the time of the suicide attempts. While it is unclear whether BDNF levels constitute a predisposing or precipitating risk factor for suicidal behaviour, studies should take into consideration the time intervals between attempt and BDNF measurement and aim for consistency. It is likely that associations between BDNF levels and suicidal behaviour will vary depending on when BDNF levels are assessed. Because only one of the six studies of BDNF level and attempted suicide included recent cases, no conclusions can be drawn regarding the relative strength of the association in recent as opposed to past suicide cases. Future studies should aim to measure BDNF in closer proximity to the suicide attempt in order to minimize the effects of unmeasured confounders that may be influenced by differences in time.

An additional point to consider is the varying methods of sample selection among studies. While some of the case-control studies separately recruited individuals who had made suicide attempts and compared them to non-suicidal controls [29, 34], other studies recruited individuals from a psychiatric population and retrospectively assessed their history of suicide attempts [37, 43]. Future studies should aim to separately recruit individuals who had attempted suicide and non-suicidal psychiatric controls in order to attain larger samples of individuals with suicide attempts and to increase the generalizability of the findings beyond individuals with a specific psychiatric disorder.

The meta-analysis of case-control studies of serum BDNF in individuals with suicide attempts and psychiatric controls revealed a small effect size of −0.32. The *p* value was not significant (*p* = 0.36). The high heterogeneity associated with this pooled estimate could be attributed to the diversity in the sample populations. Liang et al.’s sample consisted of patients with major depression, Huang and Lee’s sample consisted of patients with schizophrenia, and Deveci et al.’s sample consisted of individuals with suicide attempts with no major psychiatric disorder and control participants with major depression. This meta-analysis may be underpowered due to the small number of studies included and the low sample sizes in each study. Nonetheless, this is an important finding, as it suggests that individuals who attempt suicide do not have significantly altered serum BDNF levels compared to psychiatric controls.

Further research is necessary to elucidate the relationship between serum BDNF levels and suicidal behaviour, and to ascertain whether the relationship depends on the timing of measurements. Consistent definitions of suicidal behaviour, research methodology, and adjustment for important confounding factors (such as medication use, body mass index, and smoking status [18, 47, 48]) may help to produce a clearer understanding of the relationship. Currently, the evidence does not provide convincing support for an independent association between serum BDNF levels and suicidality.

### Plasma BDNF levels and attempted suicide

The three studies of plasma BDNF levels and suicidal behaviour present conflicting evidence of the relationship. Two of the three studies [30, 31] found significant associations between plasma BDNF levels and attempted suicide, while the third [45] did not. It is interesting to note that two studies with very similar study designs [30, 45], in which patients with depression who were hospitalized for recent suicide attempts were compared to hospitalized non-suicidal patients with depression and healthy controls, had opposing findings. Kim et al.’s 2007 study found significantly low BDNF levels in suicidal individuals compared to both control groups, but Lee and Kim’s study in 2009 found no relationship between BDNF and suicidal behaviour. The inconsistency in findings could be due to a number of factors. In all three of these studies, univariate analyses were used to compare BDNF levels among groups. While participants were matched on some variables (age and sex), no variables were adjusted for in the analyses. In addition, the sample sizes of these three studies are small; the group of individuals with suicide attempts varied from 20 to 32 individuals. Future studies should be conducted using larger samples, and using statistical analyses that adjust for confounding variables such as medication use, body mass index, and smoking status [18, 47, 48].

Another consideration is that, like in the studies of serum BDNF levels, these studies vary in the time periods between BDNF measurement and suicide attempt. Both Kim et al.’s and Lee and Kim’s studies included individuals hospitalized for recent suicide attempts, while Lee’s study included individuals with a lifetime history of suicide attempts. However, this does not explain the differences in findings, since the inclusion of recent vs. past suicide cases did not determine whether a significant association was found between BDNF level and suicidal behaviour.

Seeing that these three studies were all conducted at a single research centre in Korea, and may not have included independent samples, additional studies conducted in other locations with diverse sample populations will contribute valuably to the literature.

As of yet, the studies of plasma BDNF levels and suicidal behaviour are few in number, inconsistent in their findings, and subject to methodological limitations. No conclusions can be drawn from the existing evidence on the association between plasma levels of BDNF and attempted suicide.

### GRADE quality of evidence

While the protocol for this systematic review stated that the Grading of Recommendations, Assessment, and Evaluation (GRADE) framework would be used to report the quality of evidence, it was deemed unnecessary to do so. The GRADE framework provides a systematic approach to consider and report risk of bias, imprecision, inconsistency, indirectness of study results, and publication bias. The GRADE framework is used to summarize and evaluate the evidence according to outcome, and is useful when the results of the studies have been combined statistically. Seeing as only 3 of the 14 included studies were pooled in a meta-analysis, it was not possible to evaluate the quality of the evidence using this framework. Furthermore, the GRADE framework is best suited to summaries of randomized controlled trials and is rarely used for observational studies such as these.

## Conclusions

This is the first systematic review to explore associations between BDNF levels and suicidal behaviour. The meta-analysis of studies examining serum BDNF levels and attempted suicide revealed no significant association. The qualitative review of the literature revealed that the current evidence does not provide consistent support for an association between BDNF and suicidal behaviour. The findings of this systematic review are not in accordance with the hypothesis that lower levels of BDNF are linked to suicidal behaviour. It is possible than an association exists in parts of the brain and bloodstream, but the studies vary substantially in their methods and results, making it difficult to draw sound conclusions. The studies are also subject to a number of methodological limitations. As of yet, the studies conducted are few in number and have high risk of bias. Moreover, distinguishing the role of BDNF in suicidal behaviour from its role in mental illness is a key difficulty across studies. As this is a relatively new area of research, currently the evidence does not warrant using measures of BDNF in a clinical setting to assess suicide risk. Further studies that are well-powered, include psychiatric comparator groups, and adjust for important confounders will help to elucidate this relationship and may provide valuable information to clinicians and researchers.

## Additional files

Additional file 1:
**PRISMA checklist.** (DOC 62.0 kb) Additional file 2:
**Risk of bias assessment table.** (DOCX 14.4 kb)

## Abbreviations

BDNF: brain-derived neurotrophic factor
CSF: cerebrospinal fluid
MINI: Mini International Neuropsychiatric Interview
NOS: Newcastle-Ottawa Scale
SMD: standardized mean difference
SSI: Scale for Suicidal Ideation
PMI: Postmortem interval
