# Supplementary material for: Association between BDNF levels and suicidal behaviour: a systematic review and meta-analysis
Source: Syst Rev. 2015 Dec 30;4:187. doi: 10.1186/s13643-015-0179-z (PMC4697315; doi:10.1186/s13643-015-0179-z)
Supplement: Additional file 2: — Risk of bias assessment table. (DOCX 14.4 kb) [file 13643_2015_179_MOESM2_ESM.docx]

Table S1: Risk of Bias Assessment using the Newcastle Ottawa Scale

|  | Banerjee 2003 | Dwivedi 2003 | Karege 2005 | Maheu 2013 | Deveci 2007 | Grah 2014 | Huang 2006 | Liang 2012 | Park 2014 | Pinheiro 2012 | Kim 2007 | Lee 2007 | Lee 2009 | Martinez 2012 |
| --- | --- | --- | --- | --- | --- | --- | --- | --- | --- | --- | --- | --- | --- | --- |
| Is the source population appropriate and representative of the population of interest? | 3 | 3 | 3 | 3 | 1 | 3 | 3 | 3 | 2 | 3 | 3 | 3 | 3 | 2 |
| Is the sample size adequate and is there sufficient power to detect a meaningful difference in the outcome of interest? | 1 | 1 | 1 | 1 | 0 | 2 | 0 | 1 | 1 | 1 | 1 | 2 | 1 | 1 |
| Did the study identify and adjust for any variables or confounders that may influence the outcome? | 0 | 0 | 1 | 1 | 0 | 1 | 0 | 0 | 0 | 1 | 0 | 0 | 0 | 0 |
| Did the study use appropriate statistical analysis methods relative to the outcome of interest? | 1 | 1 | 1 | 1 | 1 | 3 | 1 | 1 | 1 | 3 | 1 | 1 | 1 | 1 |
| Is there little missing data and did the study handle it accordingly? | 3 | 3 | 3 | 3 | 2 | 3 | 2 | 3 | 1 | 3 | 3 | 3 | 3 | 3 |
| Is the methodology of the outcome measurement explicitly stated and is it appropriate? | 3 | 3 | 3 | 3 | 2 | 3 | 1 | 3 | 2 | 3 | 3 | 1 | 3 | 3 |
| Is there an objective assessment of the outcome of interest? | 3 | 3 | 3 | 3 | 3 | 3 | 1 | 3 | 2 | 3 | 3 | 1 | 3 | 3 |
